# Specific recognition of reproductive parasite workers by nest-entrance guards in the bumble bee *Bombus terrestris*

**DOI:** 10.1186/1742-9994-10-74

**Published:** 2013-12-10

**Authors:** Pierre Blacher, Laurie Boreggio, Chloé Leroy, Paul Devienne, Nicolas Châline, Stéphane Chameron

**Affiliations:** 1 Laboratoire d’Ethologie Expérimentale et Comparée E.A. 4443, Université Paris 13, Sorbonne Paris Cité, 93430 Villetaneuse, France; 2 Departamento de Biologia, FFCLRP, Universidade de São Paulo (USP), Ribeirão Preto, SP, Brazil

**Keywords:** Intraspecific social parasitism, Guarding behaviour, Honey bee, Social recognition, Defence strategy, Polistes, Coevolution, Drifting

## Abstract

**Background:**

The impact of social parasites on their hosts’ fitness is a strong selective pressure that can lead to the evolution of adapted defence strategies. Guarding the nest to prevent the intrusion of parasites is a widespread response of host species. If absolute rejection of strangers provides the best protection against parasites, more fine-tuned strategies can prove more adaptive. Guarding is indeed costly and not all strangers constitute a real threat. That is particularly true for worker reproductive parasitism in social insects since only a fraction of non-nestmate visitors, the fertile ones, can readily engage in parasitic reproduction. Guards should thus be more restrictive towards fertile than sterile non-nestmate workers. We here tested this hypothesis by examining the reaction of nest-entrance guards towards nestmate and non-nestmate workers with varying fertility levels in the bumble bee *Bombus terrestris*. Because social recognition in social insects mainly relies on cuticular lipids (CLs), chemical analysis was also conducted to examine whether workers’ CLs could convey the relevant information upon which guards could base their decision. We thus aimed to determine whether an adapted defensive strategy to worker reproductive parasitism has evolved in *B. terrestris* colonies.

**Results:**

Chemical analysis revealed that the cuticular chemical profiles of workers encode information about both their colony membership and their current fertility, therefore providing potential recognition cues for a suitable adjustment of the guards’ defensive decisions. We found that guards were similarly tolerant towards sterile non-nestmate workers than towards nestmate workers. However, as predicted, guards responded more aggressively towards fertile non-nestmates.

**Conclusion:**

Our results show that *B. terrestris* guards discriminate non-nestmates that differ in their reproductive potential and respond more strongly to the individuals that are a greatest threat for the colony. Cuticular hydrocarbons are the probable cues underlying the specific recognition of reproductive parasites, with the specific profile of highly fertile bees eliciting the agonistic response when combined with non-colony membership information. Our study therefore provides a first piece of empirical evidence supporting the hypothesis that an adapted defensive strategy against worker reproductive parasitism exists in *B. terrestris* colonies.

## Introduction

One of the most ubiquitous features of group-living species is the existence of complex recognition systems allowing individuals to precisely adjust their behaviour in a way that enhances both individual and group fitness. Elaborated recognition abilities commonly evolved in social groups because they facilitate cooperation amongst group members [1,2] and help maintain group integrity by decreasing the detrimental impact of competition, predation and social parasitism [3-6].

In social parasitism, the parasite diverts the workforce (e.g. brood care behaviour) and/or the valuable resources of a society for its own reproductive effort, thereby reducing the host’s reproductive success [7]. As a consequence parasites exert strong selection pressures upon their host which are thus likely to evolve defensive strategies to reduce the likelihood and/or the costs of being parasitized [7-9]. Guarding the nest to prevent the intrusion of parasites is a widespread response of host species (e.g. [3,6]). When absolute rejection of strangers would provide the best protection against parasites, more fine-tuned strategies could prove less costly since (1) not all strangers constitute a real threat and (2) rejection behaviours involve time losses and energy expenditure as well as the risk of injury or death (e.g. [6,10,11]). The hosts’ ability to discriminate potential parasites from non-parasites would help to refine anti-parasitic strategies. The impact of host-parasite interaction on recognition mechanisms has been the topic of many theoretical and empirical studies (e.g. [12,13]). More surprisingly, the specificity of the guarding response has rarely been tested. Most empirical researches concern birds in which the existence of an enemy-specific defensive strategy against brood-parasites has been uncovered in some species [6,14] as, for instance, in the yellow warblers *Dendroicu petechia* which displays distinct defensive patterns towards cowbirds, predators and nonthreatening intruders [14].

Social insect colonies contain valuable resources (food and workforce) and are thus the target of social parasites that attempt to invade and exploit the nest. For instance, bumble bees colonies are targeted by heterospecific social parasites such as the cuckoo bumble bees that exploit the colony workforce after having killed the host colony’s queen [15]. Exploitation by conspecific laying workers or by food-robbers is another widespread form of social parasitism [16,17]. To defend their nest against intruders, cavity-nesting species commonly deploy nest-entrance guards that actively inspect workers that attempt to enter the colony [18-21]. When detected, non-nestmates can then be harassed and repelled, therefore reducing the rate at which colonies are parasitized. Nestmate recognition in social insects involves the comparison of perceived chemical cues to an internal ‘template’ of the own colony odour, usually acquired during the days following emergence and updated during adult life ([22,23] but see [24]). Individuals belonging to the same colony indeed share a common odour, which usually consists of a specific blend of cuticular lipids whose relative proportions vary among colonies [25,26]. The agonistic response usually occurs when the dissimilarity between the chemical profile of the inspected individual and the template exceeds a given tolerance threshold, which is tuned by the evolutionary outcome of non-nestmate acceptance and nestmate rejection [27]. Downs & Ratnieks [28] showed for instance that social tolerance of *Apis mellifera* guards to conspecific intruders actually varies according to colony resources.

Efficient protection of the colony resources from robbers can be based exclusively on nestmate recognition, since all intruders entail the same potential cost to the colony. When it comes to avoid worker reproductive parasitism, things get a little more complex. The propensity of intruding workers to readily behave as parasitic egg-layers is indeed conditioned by their reproductive status at the time they join the host colony (e.g. [29,30]): while fertile intruders can directly engage in reproduction, sterile workers cannot. One could thus hypothesize that guard should be more restrictive to fertile than to sterile non-nestmates. Such fertility-based discrimination could be based on chemical cues, since cuticular lipids have been shown to reliably mirror ovary activation in most social insects species so far investigated [31]. Workers have been proved to perceive and adjust their behaviour to fertility signals in various contexts of intracolonial regulation of reproduction (i.e. policing and reproductive hierarchy, e.g. [31-33]). Specific fertility-based discrimination of potential parasites by guards has explicitly been investigated in two Apis species, *A. mellifera* and *A. cerana*[34-36]. Overall, honeybee colonies were found to adjust their social tolerance threshold to the risk of worker reproductive parasitism, but no specific discrimination of fertile individuals could be evidenced [36].

Bombus and Apis are phylogenetically closely-related genera [37] in which both worker reproductive parasitism widely occurs [16]. By contrast, while food-robbing is frequent and constitutes a major threat that can lead to the death of the targeted colony in honeybees [38], steal of honey between neighbouring colonies appears to be very rare in bumblebees [19]. In *Bombus terrestris* especially, reproductive parasitism occurs despite the presence of guards [15,19] and some evidence of nestmate recognition [19,39]. Colonies are regularly visited by conspecific non-nestmate workers [30,40,41] of which a fraction only, the fertile ones, engage in selfish reproduction [29,30]. *B. terrestris* is thus particularly adequate to investigate the specific link between guarding behaviour and reproductive parasitism avoidance, since the potential cost of an intruder mainly relies on its reproductive potential. Moreover, infertile incomers could have a non-null, positive impact on the colony fitness as non-nestmate workers take part in maintenance activities of their host colonies [29,30,40]. Therefore, since discriminating fertile from infertile visitors can significantly impact colony fitness, we predict the evolution of both colonial and fertility discrimination abilities in *B. terrestris* to allow for optimal guarding behaviour.

We here investigated the impact of both colonial origin and fertility level of conspecifics on *B. terrestris* guarding behaviour by conducting behavioural assays between guards and nestmate or non-nestmate workers of varying level of fertility in free-living colonies. Dyadic encounters were performed at the nest entrance since agonistic response is known to greatly vary depending on the context [42,43]. We predicted that guards would behave more agonistically towards non-nestmates than nestmates, and among the former towards fertile than sterile workers. Because social recognition is thought to mainly rely on chemical cues in social insects (but see [44,45]), we also conducted chemical analyses to examine whether *B. terrestris* cuticular lipids could convey reliable information about both colony membership and ovarian activity, upon which guards could suitably adjust their defensive decision. Overall, our study aimed to investigate the presence of specific recognition and behavioural discrimination of parasite workers by guards that could underlie an adapted defensive strategy to worker reproductive parasitism in *B. terrestris* colonies.

## Results

### Chemical analyses

#### General characteristics

In accordance with previous descriptions of chemical compounds found on the workers’ cuticle and in the Dufour’s gland [46-48], we detected the presence of hydrocarbons (branched methylalkanes, linear alkanes, alkenes and alkadienes), esters of fatty acids and ketones in the cuticle surface extracts. Only the cuticular lipids (CLs) that represented more than 0.1% of the variance in at least one group were retained for the analysis (see Additional file 1). These forty-five compounds were hydrocarbons (alkanes, alkenes and alkadienes) and esters whose chain length ranged from C21 to C36 (relative proportions ± sd of 66.6 ± 9.9%, 24.0 ± 8.5%, 9.6 ± 2.7% and 1.3 ± 0.8% respectively).

#### Colony membership

The discriminant analysis (DA) significantly differentiated the chemical profiles of *B. terrestris* workers according to their colonial origin (Wilks’ λ = 0.0185, F_152,297_ = 3.36, p < 0.0001, see Additional file 2). Four discriminant functions added significantly to the discrimination between the colonies (function 1: 45.0% of the variance, Wilks’ λ = 0.018, *χ*^2^ = 372.7, df = 152, p < 0.001; function 2: 21.4% of the variance, Wilks’ λ = 0.079, *χ*^2^ = 236.5, df = 111, p < 0.001; function 3: 20.1% of the variance, Wilks’ λ = 0.2, *χ*^2^ = 148.5, df = 72, p < 0.001; function 4: 13.4% of the variance, Wilks’ λ = 0.50, *χ*^2^ = 63.8, df = 35, p = 0.002). On the 116 individuals plotted on this analysis (originating from 5 colonies), 9 were not correctly assigned to their colony (92.2% of correct classification). The total amount of compounds was found to be similar in guard and introduced bees (59.3 ± 3.0 μg, n = 49 and 66.4 ± 4.3 μg, n = 67 respectively, one-way ANOVAs with ovarian development as covariant in the analysis, F_1,113_ = 0.47, p = 0.49). Our analyses therefore indicate that (1) *B. terrestris* workers bear a chemical signature specific of the colony and (2) the isolation in groups of three did not alter the colony-specific signature.

#### Reproductive status

**Figure 1:**
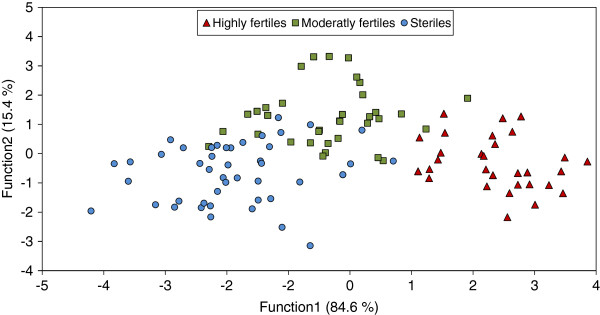
**Discriminant analysis.** Discriminant analysis of 116 *Bombus terrestris* workers based on the 38 cuticular lipids retained for the analysis, showing discrimination among three classes of ovarian development. The percentages of variance explained by each of the two discriminant functions are provided on the axis labels.

The discriminant analysis (DA) significantly discriminated the 3 classes of ovarian development (Wilks’ λ = 0.124, F_76,152_ = 3.67, p < 0.0001; 89.7% of all the individuals were correctly classified). The three classes of ovarian development were segregated (Figure 1; 1: steriles, 2: moderately fertiles, 3: highly fertiles; Mahalanobis distances, 1–3 = 21.0, F_38,76_ = 7.44, p < 0.001; 1–2 = 5.79, F_38,76_ = 2.01, p = 0.004; 2–3 = 12.12, F_38,76_ = 3.8, p < 0.001) along the first factor axis which explained 84.6% of the variance (function 1: 84.6% of the variance, Wilks’ λ = 0.12, *χ*^2^ = 197.1, df = 76, p < 0.001; function 2: 15.4% of the variance, Wilks’ λ = 0.59, *χ*^2^ = 49.4, df = 37, p = 0.082). The misclassification mostly occurred in the “moderatly fertiles” group which is intermediate between the two other groups and none of the individuals belonging to the “steriles” group were misclassified in the “highly fertiles” group and vice versa.

Compounds that present the highest fertility-discriminating effect (Pearson’s correlation with ovarian development > 0.5) are presented in Additional file 3. These compounds are mostly linear alkanes and alkenes. The two esters retained for the analyses (see Additional file 1) are not included in the Additional file 3 owing to their weak level of correlation with ovarian development (Pearson’s r = −0.15 and −0.28 for the Hexadecyl 9-octadecenoate and Icosyl hexadecanoate respectively).

The amount of chemical compounds were found to significantly differ according to the level of ovary development in workers (one-way ANOVAs, F_2,113_ = 20.57, p < 0.0001). Quantities of cuticular compounds were found to increase with the workers’ level of ovary activation (47.61 ± 2.31 μg, n = 45, 59.09 ± 3.91 μg, n = 35 and 85.14 ± 5.92 μg, n = 36 in sterile, moderately fertile and highly fertile workers respectively, Post-hoc LSD all p < 0.028).

### Dyadic encounters

Table 1 shows the duration and the occurrence of antennating and self-grooming behaviours performed by guards during dyadic encounters. Antennal contacts were overall briefs, but a significant pattern arose where guards antennated significantly more frequently and for a longer time the introduced non-nestmates than their nestmates. A similar trend occurred in the self-grooming behaviour during the dyadic encounters where guards tended to perform this behaviour more frequently and for a longer time in the presence of non-nestmates than in the presence of nestmates, but the differences were only marginally significant.

**Table 1:** Behaviour of guards towards nestmate and non-nestmate workers

	**Behaviour**	**Nestmate (n = 55)**	**Non-nestmate (n = 56)**	***df***	***F***	***P***
Duration (s)	Antennation	3.48 ± 0.36	4.58 ± 0.43	1. 108	4.21	0.042
	Self-grooming	23.4 ± 3.18	34.2 ± 4.74	1. 108	3.69	0.057
Occurrence	Antennation	3.96 ± 0.35	4.79 ± 0.31	1. 108	4.15	0.044
	Self-grooming	7.22 ± 0.78	8.98 ± 0.72	1.108	3.65	0.062

Results are presented as mean ± SE.

**Figure 2:**
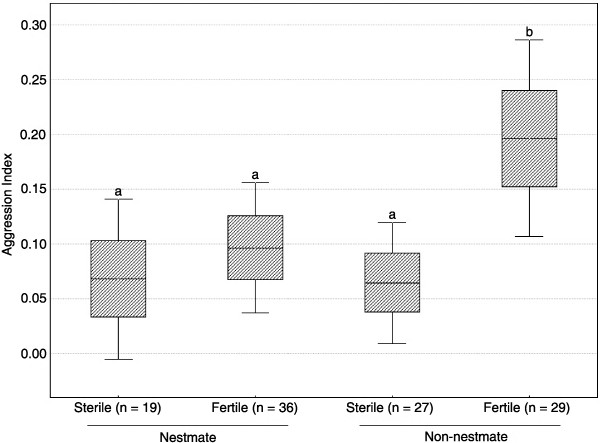
**Aggression indexes of guards during dyadic encounters according to the type of introduced workers.** Each encounter (n = 111) lasted 5 minutes. Box plots represent mean ± SE and 95% confidence interval. The different letters denote statistical differences after post-hoc REGW comparison procedure.

The aggression indexes of guards significantly differed according to the type of introduced bees (Figure 2, one-way ANOVAs, F_3,107_ = 3.69, p = 0.014). Non-nestmate fertile workers were treated significantly differently than all other worker groups (Figure 2, Post-hoc REGW comparisons all p < 0.038) and received the strongest aggressive response. Guards were similarly aggressive towards the 3 other worker groups (Figure 2; REGW all p > 0.45). The mean aggression indexes of guards were low overall (Figure 2), indicating that agonistic acts were mainly composed of threatening behaviours. Direct attacks by guards indeed occurred in 10 of the 111 encounters (9%). Interestingly, all targeted bees were fertile and 7 were non-nestmates (24.1% of fertile foreigners) vs. 3 nestmates (8.3% of fertile nestmates).

The 4 different groups of introduced bees displayed a similar aggressiveness (F_3,107_ = 1.33, p = 0.26, see Additional file 4). Guard and introduced bees exhibited a similar level of aggression scores (0.1 ± 0.02 and 0.14 ± 0.02 respectively, permutation test for paired data, p = 0.11). However, no correlation could be detected between the aggressiveness of guards and of introduced workers (r = −0.0024, p = 0.98, n = 111).

Dissections revealed that 55 of 111 (49.5%) tested guard bees had developed ovaries. The ovarian development of the guard had no significant influence neither on their aggressiveness (one-way ANOVAs, F_1,109_ = 0.23, p = 0.63) nor on the duration and occurrence of their antennating behaviour (one-way ANOVAs F_1,109_ = 1.68, p = 0.19 and F_1,109_ = 0.50, p = 0.47 respectively).

## Discussion

Identifying potential parasites at the entrance of the nest requires assessing both the colonial membership and the fertility of the intruders. We actually found that the cuticular compounds of bumble bees convey both types of information, therefore allowing the evolution of a specific defensive strategy against worker reproductive parasitism by *Bombus terrestris* guards. Behavioural analyses indeed proved that even though guard bees could discriminate nestmates from non-nestmates, only fertile intruders triggered a rise in the agonistic response.

It is well-documented in a wide range of social insect species that within-colony variations of cuticular lipid (CL) profiles correlate with fertility status [31,32,49]. Direct evidence that fertility-related compounds are actually perceived and used as fertility signals however mostly comes from studies in ants [50,51]. Here we provide compelling evidence that CLs convey valuable information about the workers’ reproductive potential in the bumble bee *B. terrestris*, as has been previously proposed by Sramkova et al. [46]. Our results indeed show that reproductive and non-reproductive workers’ cuticles bear distinct chemical patterns. More precisely, our results show that CLs reliably reflect worker fertility level, rather than crudely signalling the workers’ ability to lay eggs, since worker chemical profiles could be separated according to ovarian development classes. Amsalem et al. [47] analysed the content of the Dufour’s gland in workers of various fertility levels and reported that some esters were specific of infertile workers. They then proposed that the Dufour’s gland secretion may regulate intra-colonial dominance interactions, with sterile workers advertising that they are out of the competition. By showing that cuticular hydrocarbons covary with ovarian activity, we provide another putative chemical candidate for the recognition of competing workers in *B. terrestris*. Whether fertility or sterility signalling (or both) mediate aggressions will have to be assessed in future studies. Apart from fertility, CL profiles also allowed clear segregation of workers according to their colony of origin, indicating that they can also reliably signal group membership. In accordance with previous empirical evidence of the involvement of CLs in both nestmate recognition and fertility assessment in social insects [26,31,33,49,52-56], we therefore propose that CLs play a major role in the detection of potential intraspecific parasites by bumble bee guards.

In order to ensure a strong ethological relevance to our guarding assay, we chose to confront bees at the very nest entrance. When most studies on guards in bees follow the Downs & Ratnieks [28] procedure using cooled intruders in order to avoid flight, we took advantage of the tunnel entrance of natural *B. terrestris* nests [15,41] to ensure that guards and intruders have a high probability of contact. The counterpart is that both bees in the dyadic encounters were fully behaviourally active, thus making it more complex to decipher the proximal cause for each individual’s behaviour in the interaction. Because we could not find any difference in the intruders’ behaviour according to their colonial origin and/or fertility status, we can therefore safely conclude that the guards’ behavioural response was not a mere reaction to the intruder’s behaviour but instead genuinely relied on some social recognition mechanism.

Interestingly enough, we found that in our conditions guards were as tolerant to infertile non-nestmates as to nestmates (be they fertile or not). We however showed that non-nestmates elicited significantly more antennal contacts and marginally more self-grooming behaviours, proving that they were not mistaken for nestmates. Such a high social tolerance is in accordance with previous observations in free-living bumble bee colonies [30,40,57], while in sharp contrast with the strong colonial closure usually described in social insect colonies (see [58,59] but see [35,60]). In social insects, colony workforce is generally a crucial determinant of colony reproductive success (e.g. [61]). This may be especially true in short-lived species with small-sized colonies, such as *B. terrestris*, where fast colony development is crucial to successfully rear the sexuals before the end of the season. Worker number, colony growth rate and queens’ inclusive fitness have indeed been shown to be positively correlated in *B. terrestris*[62]. As a consequence, except if intruders remain inactive in the nest or perform costly behaviour such as egg-laying or food-robbing, welcoming foreign workers may be beneficial for *B. terrestris* colonies. Infertile *B. terrestris* workers have actually been shown not to reproduce in host colonies [29,30]. Furthermore, behavioural observations in the laboratory and semi-natural conditions have shown that they perform in-nest activities such as intense brood-rearing [29] and also regularly engage in foraging activities for their host colony [30,40]. The high tolerance of *B. terrestris* guards towards sterile foreign workers may therefore prove adaptive if the latter integrate the colony workforce and consequently enhance the host colony fitness.

By contrast, *B. terrestris* fertile non nest-mates usually engage in egg-laying [30,40] and are thus a serious potential threat for colony fitness. Moreover, reproductive intruders aggressively compete with host workers [40], which may negatively impact their propensity to develop or retain functional ovaries [63-65]. As predicted, we found that *B. terrestris* guards were more aggressive towards fertile foreign workers than towards infertile ones, thus showing the guards’ ability to specifically recognize parasite workers. Interestingly, while direct attacks towards parasite workers occurred in a fraction of the encounters (24,1%), agonistic acts were in most encounters limited to threatening behaviours, which contrasts with the fierce defence behaviour usually described in honey bees (e.g. [28]). It is indeed not unusual that honeybee guards fight non-nestmates workers to death (e.g. [66]). Such between-species differences in the level of the defensive response may stem from a differential impact of escalated fights on the colony fitness. Indeed, if the loss of individual guards consecutive to overt fights may be little costly for colonies made up of thousands of individuals, it may be nontrivial in small *B. terrestris* colonies of a few hundred individuals. As a consequence, avoiding costly fights is likely to be beneficial for both guards and intruders in *B. terrestris* colonies. As in the tiny colonies of the hover wasp *Liostenogaster flavolineata* where conspecific intruders are usually repelled with minimal contact [67], *B. terrestris* could have evolved a ritualized nest-guarding behaviour to resolve conflict without casualties [68-70]. However, such strategy could be selected for only if it efficiently prevents parasites from entering the nest or starting to reproduce within the nest (threatening behaviours usually expressed in the context of the intracolonial reproductive competition in bumblebees may induce ovary regression [30,63]), which should be assessed by further studies. Alternatively, the low intensity of the agonistic response towards parasitic workers may have stemmed from the low detrimental impact of parasitism in our large experimental colonies. Chapman et al. [35] reported that permissiveness of honey bee *Apis mellifera* colonies towards unrelated workers depends on social context, with colonies more vulnerable to parasitism, the queenless ones, being less tolerant than queenright colonies. In *B. terrestris*, parasitism by egg-laying workers is likely to be more costly in incipient colonies since uncontrolled worker reproduction may disrupt the ergonomic growth of the colony [63,71,72]. Furthermore, large colonies could be highly resistant to parasite reproduction as most eggs could readily be policed by the numerous workers of the colony [40,73]. Therefore, the potential costs of parasitism are thus likely to be a negative function of the colony size in *B. terrestris*. As in honeybees where workers display defensive behaviours from weakly to highly aggressive depending on the context [66], bumble bee guards’ agonistic response may be plastic and its intensity adjusted according to a fine balance between the costs of being parasitized and the risk for their own survival.

## Conclusion

In this study, we investigated whether *B. terrestris* have evolved a specific colony defence strategy in response to parasitism by conspecific reproductive workers. We show that bumble bee colonies adjust their defensive response to the threat level in an apparently adaptive way. We indeed found that *B. terrestris* guard workers were completely tolerant to infertile non-nestmates, probably because accepting them does not entail costs and may even have a positive impact on the colony fitness. By contrast, we showed that they were less tolerant towards workers that can actively parasitize the nest. We further demonstrated that variation in CL profiles closely reflect the current fertility of workers and their colony membership, which suggest that they could underlie the discrimination against fertile intruders. A defensive behaviour is defined as a counter-adaptation to parasitism if the agonistic response is beneficial and specific to parasites [74]. We here provide evidence of the existence of a distinctive agonistic response to intra-specific reproductive parasites in *B. terrestris* colonies. Further research on its efficiency either in preventing parasite intrusion or in controlling parasite reproduction in various host conditions (e.g. various colony sizes) would help to better characterize the adaptive value of this behaviour.

## Methods

### Colonies and rearing conditions

We used 10 *B. terrestris* colonies obtained from GTICO SARL (Villeneuve l’Archevêque, France) in Mai 2011. Colonies were received a few days after the emergence of the first workers and had a queen and brood at every developmental stage. They were reared in wooden boxes (17.5 × 26 × 15 cm) in a dark room at a temperature of 28 ± 2°C and a relative humidity of 55 ± 5%. Colonies were fed ad libitum with sugar syrup (mix of water and sugar concentrated at 64–65%) and fresh pollen.

### Experimental procedure

The experimental procedure included three phases. First, we manipulated some workers in order to constitute groups of fertile and infertile bees of the same age that could be used as intruders in bioassays. Then, we conducted dyadic encounters between these workers and nestmate and non-nestmate guards. Finally, all tested bees were dissected to check their ovarian development and chemical analysis was performed on a sample of guards and introduced bees.

#### Colony and worker preparation

**Figure 3:**
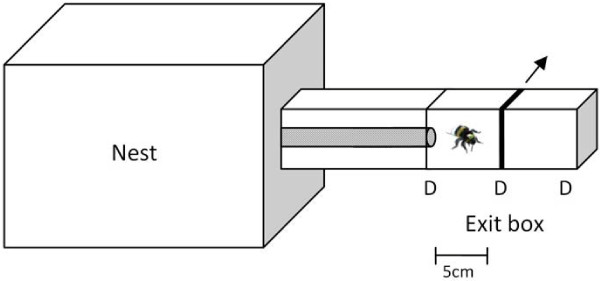
**Design of the experimental nests.** For each encounter, a guard patrolling at the entrance of the nest is trapped into the exit box of its colony. The exit box is then gently transferred into the laboratory. One experimental bee is then introduced in the box in which it is allowed to habituate to the device for 30 s. Tests begin after the microscope slide in the middle of the box is removed and the first interaction between the bees occur. Encounters are video-recorded and last five min. The letters D indicate the positions of the microscope slides allowing to close/open the different parts of the exit box.

Upon reception of the colonies, all adult bees received a colony-specific colour combination for subsequent identification of colony origin. Then, in each of the colonies, a cohort of newly emerged workers was marked with the colony-specific colours as well as with numbered tags (Opalith Plättchen, Friedrich Wienold, Germany). These workers were reintroduced into their native colony where they stayed at least three days to allow their social integration (e.g. acquisition and learning of the colonial odour). These marked bees were thereafter isolated in groups of three in separate wooden-boxes. This separation from the queen leads to the set-up of a dominance hierarchy between the 3 workers with one worker always becoming a laying-bee with developed ovaries while the two others remain with inactive ovaries [29,30,75]. From the day the groups of three were constituted, colonies were placed in an open environment on the roof of the laboratory and each nest box was connected to a plastic tube ending with an exit box (Figure 3). The microscope slides were kept open to allow workers to forage freely until the end of the experiment. Exit boxes were thus passively odour-marked [76] through the frequent returns for foraging trips.

#### Discrimination and aggression tests

Seven days after the constitution of groups of three workers, we started the bioassays that aimed to examine the impact of both colonial origin and fertility level of conspecifics on *B. terrestris* guarding behaviour. At this time of the experiment, colonies reached a size of 144.1 ± 17.7 (mean ± sd) workers. Bioassays consisted in dyadic encounters in a non-neutral arena (the exit boxes of colonies) between a guard and one of the previously isolated bees. For each encounter, a guard patrolling at the entrance of the nest was trapped into the exit box of its colony (Figure 3). We assured that the bee was patrolling for a few minutes before trapping it, since young foragers often briefly patrol the nest-entrance before performing their first foraging trip [77]. The box was then gently transferred into the laboratory. An experimental bee was removed from its group of three and introduced in the test box, in which it was allowed to habituate for 30 s. Tests began after the central microscope slide was removed (Figure 3) and the first interaction between the bees occurred. Encounters were video-recorded and lasted five minutes. A total of 111 encounters were performed with each colony acting as both donor and receiver of introduced bees for all the others. For each receiver colony, the colonial origin of the introduced bee was pseudo-randomly alternated so that (1) no more than 2 successive encounters with nestmate or non-nestmate bees and (2) no more than 2 encounters with non-nestmate workers of the same colonial origin occurred. After each test, guards and experimental bees were sacrificed by freezing and stored at −40°C pending ovarian dissections and chemical analyses.

For each encounter, we quantified the time bees spent performing the following behaviours: displacement, immobility, antennation, contact, self-grooming, buzzing (fast short wing vibrations while opposite the other bee), pumping (distinct dorsoventral pumping movements with the abdomen), front leg movements while facing the other bee, darting movements, head butting, biting and stinging attempts [78-80]. The video analyses were done twice, once focusing on the guard and the other on the introduced bee; they were carried out in random order and under ‘blind’ conditions for the observer.

Behaviours were classified in 3 categories according to their level of aggressiveness [63,78-80]. Antennal contacts constituted the first category as it is a neutral behaviour in which bees investigate each other. The threatening behaviours constituted the second category and included buzzing, pumping and front leg movements. Overt aggression constituted the third category and included darting movements, head butting, biting and stinging attempts. We calculated an aggression index (AI) by adapting the scores proposed by Hefetz et al. [81] and Errard & Hefetz [82] and used the following values for the respective behaviour: 0, *antennation*; 1, *threat*; 2, *overt aggression*. The overall aggression exhibited by each worker (AI) was calculated as follow,

$$\mathrm{AI}=\frac{\sum_{i=1}^{n}\mathrm{AIi}*\mathrm{ti}}{T}$$

where AI_i_ represents the index of aggression, t_i_, the duration of each act and T, the total interaction time defined as the sum of durations in which the bees were interacting.

#### Fertility measurement

All the tested bees (guards and intruders) were dissected and the mean size of the eight terminal oocytes was used to assess ovarian development. We classified bees as being fertile or sterile if their ovarian index was higher or lower than 1.4 mm, respectively.

For chemical analyses, an intermediate class of ovarian development was created in order to precisely investigate whether cuticular lipids reliably reflect fertility in workers. Individuals were thus assigned to three ovarian developmental classes: (1) undeveloped ovaries (< 1.4 mm; Mean ± SD of 0.008 ± 0.08 mature eggs); (2) moderately developed ovaries (between 1.4 and 2.5 mm; Mean ± SD of 1.39 ± 1.24 mature eggs); (3) fully developed ovaries (> 2.5 mm; Mean ± SD of 5.80 ± 2.13 mature eggs).

#### Chemical analyses

Cuticular compound extraction was performed on 116 workers comprising 21–27 workers in each of the five randomly chosen colonies. For each colony, the sample of workers comprised (1) at least 8 guards and 8 introduced bees and (2) a similar number of workers in each of the 3 ovarian development classes for both guards and introduced bees. We thus retained for the analysis 45 workers in the undeveloped ovary class, 37 in the moderately developed ovary class and 34 in the fully developed ovary class. According to Oldham et al. [83] and our own preliminary investigation on thoraxes, legs, wings and antennae samples, chemical profiles are constant over the whole body in *B. terrestris* workers. We used legs to sample cuticle surface compounds in order to avoid possible contamination by the paint marking or the Dufour gland during extraction. This allowed putting aside the abdomens for ovary dissections. The cuticular compounds of bees were extracted by immersing five legs in 200 μl of pentane for 20 min. An internal standard (C_18_) was added to each extract. Extracts were dried under nitrogen and then dissolved again in 50 μl of pentane; two microliters were analysed on Agilent 7890A gas-chromatograph, equipped with an HP- 5MS capillary column (30 m × 250 μm, 0.25 μm thickness) and a split-splitless injector, coupled to a 5975 Agilent Mass Spectrometer operated at 70 eV in the electron impact ionization mode. The carrier gas was helium at 1 ml.min-1. The column oven was programmed as follows: an initial hold of 1 min at 70°C, then increased to 220°C at 30°C.min − 1, to 300°C at 4°C.min − 1, and then to 320°C at 20°C.min − 1 (held for 5 min).

Of the 79 peaks detected in our preliminary analyses, those representing more than 0.1% in at least one of the group (colonies and ovarian development classes) were retained for the analyses. Forty-five cuticular lipids (CLs) were included in the analyses. These compounds were identified on the basis of mass spectra and their retention times, compared with standard linear hydrocarbons as well as with the published literature of chemical compounds found in *B. terrestris*[46-48]. The peak areas were integrated by Agilent Chemstation software, and the relative proportion of each peak was calculated.

### Statistical analyses

For chemical analyses, peak areas were first transformed according to Z = arcsine (√Ap/100), where Z is the standardized peak area and Ap is the peak area, in order to approach normality and reduce the heterogeneity of the variance [84]. Multivariate analyses were performed using the chemical variables from the standardized data set to determine whether variation in CLs allows one to differentiate among individuals according to their colony of origin and reproductive status. In order to avoid multicollinerarity problems and unstable results in multivariate methods [85,86], a correlation matrix was computed and the peaks that were highly correlated (r^2^ ≥ 0.7, see [86]) were removed prior to analyses (see Additional file 1). For colonial origin, a standard discriminant analysis (DA) was performed to estimate the divergence (or similarity) of the chemical profiles of the different colonies and whether isolated bees could be correctly classified with the guards to their natal colony. To investigate whether CL profiles reflect the current workers’ fertility, we performed a discriminant analysis (DA) on workers of the 3 different ovarian classes. Total amount of compounds on worker’s cuticle were compared using one-way ANOVAs. Relative proportions of compounds according to the 3 different ovarian classes were compared using one-way ANOVAs followed by Least Significant Difference (LSD) post-hoc tests. Pearson’s correlation coefficients between the relative proportion of compounds and the index of ovarian development were then calculated. We considered a compound to have a high fertility-discriminating effect when the Pearson’s r was below −0.5 or above 0.5.

For behavioural analysis, we compared the occurrence and duration of antennation and self-grooming behaviours performed by guards in the various type of encounters using factorial ANOVAs with colony membership (nestmate and non-nestmate) and fertility level (fertile and infertile) of introduced bees as factors. When necessary, Box-Cox transformation was applied to achieve normality [87]. The influence of the ovary state on behaviour was tested using one-way ANOVAs. Aggression indexes of guards towards the different types of introduced workers (i.e. nestmate sterile, nestmate fertile, non-nestmate sterile and non-nestmate fertile) were compared using one-way ANOVAs followed by Ryan-Einot-Gabriel-Welsch (REGW) post-hoc comparison procedure adapted to unequal sample sized [88]. Indexes were transformed using arcsine-square-root transformation [89] prior to parametric analyses to achieve homoscedasticity. Aggression indexes of guards and introduced bees were compared using permutation test for paired data. Finally, the correlation between aggressiveness of guards and introduced workers was calculated using Pearson’s correlation coefficients.

Parametric statistical tests were done using Statistica v8.0 (Statsoft, 2007) and R-2.15.0 [90] (with the package mutoss) whereas non-parametric statistical tests were computed using StatXact-8 (Cytel Software Corporation, Cambridge, MA, USA). The level of significance was set at P ≤ 0.05. All results are stated as Mean ± SE.

## Abbreviations

CL: Cuticular lipid.

## Competing interests

The authors declare that they have no competing interests.

## Authors’ contribution

PB, SC and NC conceived the study. PB performed statistical, chemical and behavioural analysis, participated in the carrying out of behavioural assays and wrote the manuscript. LB participated in the carrying out of behavioural assays, in the behavioural analysis and helped to build the experimental set-up. CL carried out the GC-MS analysis and identified chemical compounds. PD worked out the experimental set-up. SC and NC helped to draft the manuscript. All authors read and approved the final manuscript.

## Supplementary Material

Additional file 1 **Cuticular lipids retained for the analyses.** * peaks 5, 7, 15, 21, 23, 27 and 33 were excluded of multivariate analyses because of their high level of correlation (r^2^ > 0.7) with at least one other compound, see ‘Statistical analyses’ section. Click here for file

Additional file 2 **Discriminant analysis.** Discriminant analysis of 116 *Bombus terrestris* workers based on the 38 cuticular lipids retained for the analysis, showing discrimination among five colonies. Each colony is represented by a different colour and symbol combination. The percentages of variance explained by each of the three discriminant functions are provided on the axis labels. Click here for file

Additional file 3 **Relative proportions of the compounds with the highest fertility-discriminating effect according to workers’ fertility level.** Pearson’s r correlations are calculated between the ovarian indexes and the relative proportions of compounds in workers (all p < 0.001). Relative proportions of each compound are significantly different in workers of different fertility levels (one-way ANOVAS, all p < 0.0001; LSD post-hoc tests, all p < 0.05). Results are presented as mean ± SE. Click here for file

Additional file 4 **Aggression indexes of the 4 different groups of introduced workers.** Each encounter (n = 111) lasted 5 minutes. Box plots represent mean ± SE and 95% confidence interval. The different groups displayed a similar aggressiveness (F_3,107_ = 1.33, p = 0.26). Click here for file
