# Supplementary material for: Specific recognition of reproductive parasite workers by nest-entrance guards in the bumble bee Bombus terrestris
Source: Front Zool. 2013 Dec 10;10:74. doi: 10.1186/1742-9994-10-74 (PMC3878879; doi:10.1186/1742-9994-10-74)
Supplement: Additional file 1 — Cuticular lipids retained for the analyses. * peaks 5, 7, 15, 21, 23, 27 and 33 were excluded of multivariate analyses because of their high level of correlation (r2 > 0.7) with at least one other compound, see ‘Statistical analyses’ section. [file 1742-9994-10-74-S1.pdf]

Peak no.	Compound	Peak no.	Compound
1	Heneicosane	21-23	Nonacosene (3 isomers)*
2	Docosane	24	Nonacosane
3	Tricosene	25-27	Triacontene (3 isomers)*
4	Tricosane	28	Triacontane
5	Tetracosane *	29-32	Hentriacontadiene (4 isomers)
6-7	Pentacosene (2 isomers) *	33-34	Hentriacontene (2 isomers)*
8	Pentacosane	35	Hentriacontane
9	Hexacosane	36	Dotriacontene
10-13	Heptacosene (4 isomers)	37-40	Tritriacontadiene (4 isomers)
14	Heptacosane	41-43	Pentatriacontadiene (3 isomers)
15-16	Octacosene (2 isomers) *	44	Hexadecyl 9-octadecenoate
17	Octacosane	45	Icosyl hexadecanoate
18-20	Nonacosadiene (3 isomers)		
