# Supplementary figures and images for: Specific recognition of reproductive parasite workers by nest-entrance guards in the bumble bee Bombus terrestris
Source: Front Zool. 2013 Dec 10;10:74. doi: 10.1186/1742-9994-10-74 (PMC3878879; doi:10.1186/1742-9994-10-74)

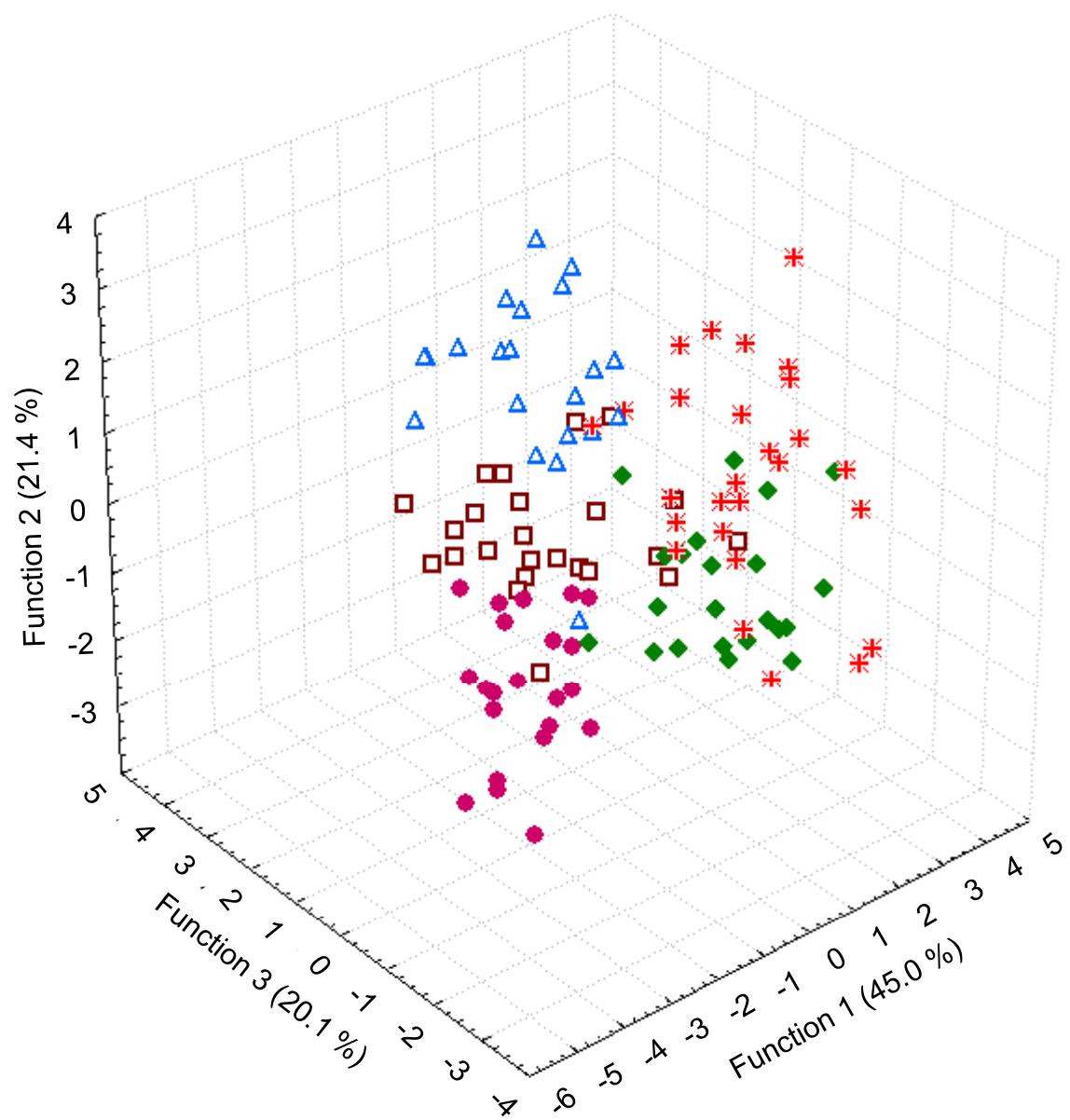

Supplement: Additional file 2 — Discriminant analysis. Discriminant analysis of 116 Bombus terrestris workers based on the 38 cuticular lipids retained for the analysis, showing discrimination among five colonies. Each colony is represented by a different colour and symbol combination. The percentages of variance explained by each of the three discriminant functions are provided on the axis labels. [file 1742-9994-10-74-S2.pdf]

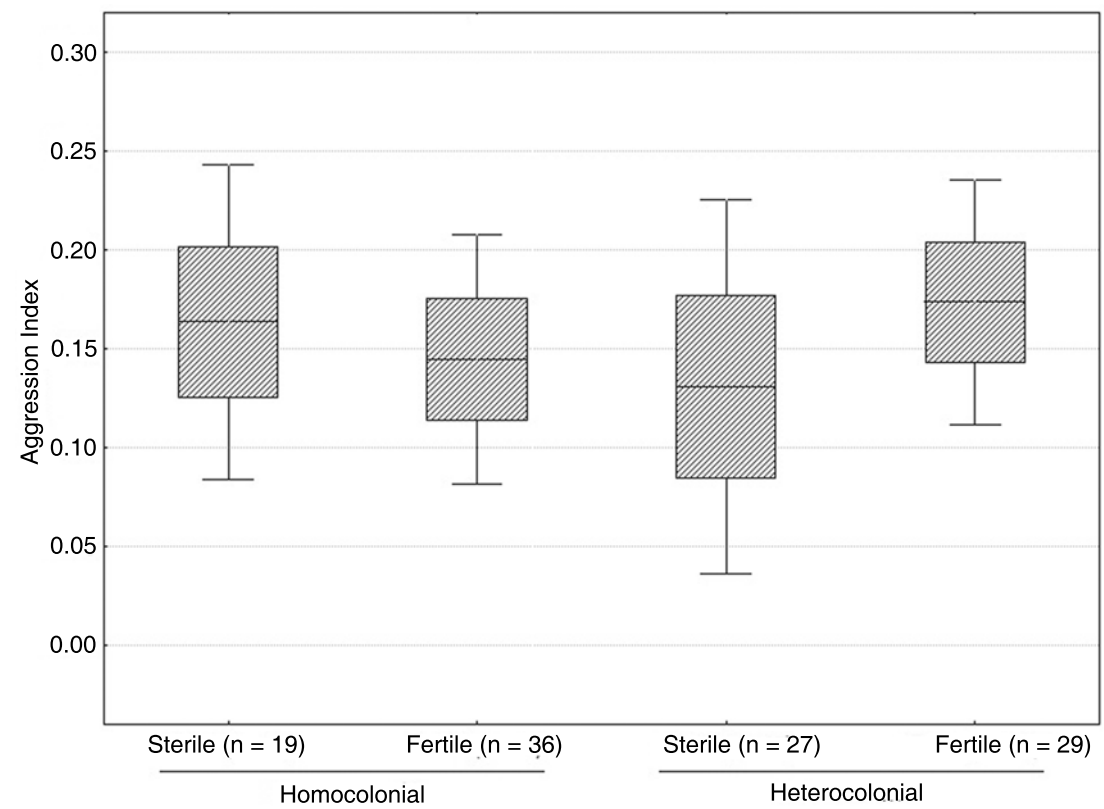

Supplement: Additional file 4 — Aggression indexes of the 4 different groups of introduced workers. Each encounter (n = 111) lasted 5 minutes. Box plots represent mean ± SE and 95% confidence interval. The different groups displayed a similar aggressiveness (F3,107 = 1.33, p = 0.26). [file 1742-9994-10-74-S4.pdf]
