# Supplementary material for: Specific recognition of reproductive parasite workers by nest-entrance guards in the bumble bee Bombus terrestris
Source: Front Zool. 2013 Dec 10;10:74. doi: 10.1186/1742-9994-10-74 (PMC3878879; doi:10.1186/1742-9994-10-74)
Supplement: Additional file 3 — Relative proportions of the compounds with the highest fertility-discriminating effect according to workers’ fertility level. Pearson’s r correlations are calculated between the ovarian indexes and the relative proportions of compounds in workers (all p < 0.001). Relative proportions of each compound are significantly different in workers of different fertility levels (one-way ANOVAS, all p < 0.0001; LSD post-hoc tests, all p < 0.05). Results are presented as mean ± SE. [file 1742-9994-10-74-S3.pdf]

compound	workers' ovarian development			Pearson's r
	low (n = 45)	medium (n = 35)	high (n=36)	
tetracosane	0.42 ± 0.02	0.49 ± 0.02	0.70 ± 0.03	0.63
pentacosane	13.77 ± 0.68	16.26 ± 0.63	22.17 ± 0.99	0.62
hexacosane	0.88 ± 0.04	1.05 ± 0.05	1.28 ± 0.03	0.61
heptacosene (four isomers)	1.69 ± 0.08	1.35 ± 0.11	0.93 ± 0.04	-0.55
octacosene (two isomers)	0.41 ± 0.02	0.29 ± 0.02	0.17 ± 0.01	-0.69
nonacosene (three isomers)	13.94 ± 0.62	9.76 ± 0.67	5.31 ± 0.22	-0.75
triacontene (three isomers)	0.80 ± 0.02	0.70 ± 0.03	0.47 ± 0.02	-0.64
hentriacontene (two isomers)	12.63 ± 0.41	10.98 ± 0.40	8.23 ± 0.38	-0.61
pentatriacontadiene (two isomers)	0.15 ± 0.01	0.27 ± 0.03	0.42 ± 0.02	0.67
